# A Novel Data Sampling Driven Kalman Filter Is Designed by Combining the Characteristic Sampling of UKF and the Random Sampling of EnKF

**DOI:** 10.3390/s22041343

**Published:** 2022-02-10

**Authors:** Tipo Cui, Xiaohui Sun, Chenglin Wen

**Affiliations:** 1 School of Automation, Hangzhou Dianzi University, Hangzhou 310018, China; cuitipo@hdu.edu.cn (T.C.); sun_xh1993@hdu.edu.cn (X.S.); 2 School of Automation, Guangdong University of Petrochemical Technology, Maoming 525000, China

**Keywords:** novel Kalman filter, large sample ensemble, EnKF, UKF, data ensemble centroid

## Abstract

In order to improve the performance of the Kalman filter for nonlinear systems, this paper contains the advantages of UKF statistical sampling and EnKF random sampling, respectively, and establishes a new design method of sampling a driven Kalman filter in order to overcome the shortcomings of UKF and EnKF. Firstly, a new sampling mechanism is proposed. Based on sigma sampling with UKF statistical constraints, random sampling similar to EnKF is carried out around each sampling point, so as to obtain a large sample data ensemble that can better describe the characteristics of the system variables to be evaluated. Secondly, by analyzing the spatial distribution characteristics of the obtained large sample ensemble, a sample weight selection and assignment mechanism with the centroid of the data ensemble as the optimization goal are established. Thirdly, a new Kalman filter driven by large data sample ensemble is established. Finally, the effectiveness of the new filter is verified by computer numerical simulation experiments.

## 1. Introduction

Nonlinear system filtering has always been a difficult problem in the field of target tracking. The extended Kalman filter (EKF), unscented Kalman filter (UKF) and other filtering methods are often used in the application fields of Unmanned Aerial Vehicle (UAV) attitude estimation, autopilot, radar detection and so on. In these fields, the improvement of filtering accuracy will have a great impact on practical applications [1,2,3,4,5,6]. Although there are many filtering algorithms that can be used in nonlinear systems, such as EKF [7], UKF [8], particle filter (PF) [9], etc., though these filtering methods have their limitations. The principle of the EKF algorithm is to linearize a non-linear system, in which only the first-order terms of Taylor expansion are preserved. For non-linear systems, retaining only the first-order terms can result in a very large loss of filtering accuracy. In high order strongly nonlinear systems, it is more difficult to linearize the system by calculating the Jacobian matrix. The error accumulation of error covariance matrix during linearization also results in a loss of filtering accuracy. In actual situations, most of the systems we encounter are nonlinear and non-Gaussian systems. The premise of the EKF algorithm is that the noise of the system is Gaussian noise [7].

The core of the UKF algorithm is the UT transformation, hich uses an ensemble of sigma sampling points to approximate the nonlinear system model [10,11,12]. The computational effort of the UKF algorithm is the same as that of the EKF, and the filtering accuracy is better than that of the EKF, which can at least reach the second-order accuracy of Taylor expansion. However, the shortcomings of the UKF algorithm are also obvious. It can only be applied to Gaussian systems, and the problem of non-positive definite error covariance matrix will be caused by the emergence of negative weight in the implementation of the algorithm, and the frequency of this problem will be greater as the order of the system increases and the nonlinearity becomes stronger. Particle filter (PF) algorithm uses the random sampling method to obtain the sample points of the state as much as possible. These sample points are used to approximate the distribution of the system as much as possible, so as to estimate the system state [9,13]. Particle filter can be applied to nonlinear non-Gaussian systems, but particle filter requires a large number of sample points, the calculation cost is too high and the problem of particle degradation occurs in the calculation process, which makes the implementation of particle filter more difficult [9].

Ensemble Kalman filter (EnKF) was first proposed by Geir Evensen in 1994 [14]. When this method was proposed, it was mainly used to solve the nonlinear problems in weather forecasting. In the face of nonlinear weather characteristics and huge meteorological data, it is very difficult to apply EKF directly to atmospheric prediction [15,16,17]. In order to apply Kalman filtering to atmospheric prediction, Geir Evensen introduced the idea of ensemble prediction and proposed a new filtering method. EnKF algorithm combines Kalman filtering with the ensemble prediction idea, uses the Monte Carlo method to design an ensemble of prediction states, takes the average value of the state ensemble as the best estimation value of the state and takes the sample covariance of the ensemble as the approximation of the covariance of the state estimation error [14,18]. The ensemble Kalman filter algorithm is an improvement of the Kalman filter. In fact, it adds the idea of ensemble, expands the sample points, supplements a variety of possibilities of the actual state, and essentially changes the sampling method. The random sampling method of EnKF using Monte Carlo also has its limitations [19,20,21,22,23]. Although random sampling will get statistical results, the random sampling will be affected by the uncertainty factors of current noise, which will lead to the loss of filtering accuracy. Moreover, there is no definite method to select the range of disturbance [24,25,26,27]. When calculating the best estimate, the EnKF algorithm uses the set average of states and the average weight, which will also reduce the filtering accuracy [28,29,30].

Therefore, a novel Kalman-filter-design method combining UKF and EnKF sampling mechanism is proposed in this paper. It is mainly used to solve the problems of nonlinear systems and strongly nonlinear systems. It can be applied in the fields of target tracking, marine weather prediction and so on. This method avoids the problem that the sampling set obtained by UKF sampling mechanism is not representative. It overcomes the problem that EnKF Monte Carlo random sampling cannot be constrained by the true statistical characteristics of the state. It reduces the error caused by using equal weight to obtain the set average value in EnKF. It avoids the failure of the UKF algorithm caused by improper selection of parameters. In addition, the method in this paper provides a reliable improvement direction for the UKF algorithm.

The remaining parts of this paper are organized as follows: Section 1 is the preface, which introduces the development status of filter and the method proposed in this paper; Section 2 is the description of nonlinear system; Section 3 and Section 4 analyze and introduce the advantages and disadvantages of UKF and EnKF; in Section 5, a new data sampling driven Kalman filter design method is introduced, which combines the characteristic sampling of UKF with the random sampling of EnKF; Section 6 is the performance analysis of the system; Section 7 introduces the numerical experiment of this method; and Section 8 presents a summary and prospect.

## 2. Nonlinear System Description

Considering the following multidimensional non-linear systems, the state equation and the measurement equation are both non-linear, respectively:

(1) x(k+1)=f(x(k))+w(k)

(2) z(k+1)=h(x(k+1))+v(k+1)

where $x(k)\in R^{n\times 1}$ is the n-dimensional state vector at time k and $z(k+1)\in R^{m\times 1}$ is the measure vector at time k+1. f(⋅) is the transformation operator and h(⋅) is the measurement transformation operator. w(k),v(k+1) is process noise and measurement noise, respectively, and w(k)~N(0,Q(k)), v(k+1)~N(0,R(k+1)).

Given the initial value x(0), get the estimated value ${\hat{x}}_{0}$ of the initial value

(3) $${\hat{x}}_{0}=E\{x(0)\}$$

**Remark** **1.** *If the state is one-dimensional, its expectation is equal to itself.*

Given the estimates x(0) and z(1),z(2),⋯,z(k) of the initial values, the estimates $\hat{x}(k|k)$ of the time k are obtained

(4) $$\hat{x}(k|k)=E\{x(k)|{\hat{x}}_{0},z(1),z(2),\cdots ,z(k)\}$$

**Remark** **2.**
$\hat{x}(k|k)$ *of the above equation is the estimated value of the state at time* k *, and* $\hat{x}(k+1|k)$ *is the predicted value at time* k+1 *, All of the following “* k∣k *” and “* k+1∣k *” are used for this purpose.*

Estimated error covariance P(k∣k) at time k is obtained from the state estimate $\hat{x}(k|k)$ at time k 

(5) $$P(k|k)=E\left\{\left[x(k)-\hat{x}(k|k)\right]{\left[x(k)-\hat{x}(k|k)\right]}^{T}\right\}$$

In the above way, based on initial values x(0), measurements z(1),z(2),⋯,z(k) , and systems (1) and (2). Assume that the estimated value and estimation error covariance matrix of state x(k) based on EnKF have been obtained

(6) $${\hat{x}}^{E}(k|k),P^{E}(k|k)$$

And the estimation value and estimation error covariance matrix based on UKF are also obtained

(7) $${\hat{x}}^{U}(k|k),P^{U}(k|k)$$

The superscript “E” in Equation (6) denotes the filter result of EnKF; the superscript “U” in Equation (7) denotes the filter result of UKF.

The objectives of the Section 3 and Section 4 below are to give the recursive filtering processes of EnKF and UKF after obtaining z(k+1), and to analyze their advantages and disadvantages, which will lay a foundation for further obtaining new filters.

## 3. Ensemble Kalman Filter Algorithm

The EnKF algorithm is a filtering method based on the Monte Carlo method and the ensemble prediction idea [14,15,16,17]. The main difference between the EnKF method and Kalman filter method is that it uses ensemble to expand the dimension of state, and then weights its state. Therefore, according to Equation (6), on the basis that z(k+1) has been obtained. Based on the EnKF method, the estimated value ${\hat{x}}^{E}(k+1|k+1)$ of state x(k+1) and the estimation error covariance matrix $P^{E}(k+1|k+1)$ will be derived. The specific algorithm steps are as follows:

Step 1: Based on the Monte Carlo random sampling mechanism, the sampling ensemble of state x(k) is obtained

(8) $$\left\{{\hat{x}}_{i}(k|k)|{\hat{x}}_{i}(k|k)={\hat{x}}^{E}(k|k)+r_{i}(k),i=0,1,\cdots ,L;r_{0}(k)=0\right\}$$

where, $r_{i}(k)$ is an arbitrary random perturbation, for example, a frequently chosen is $r_{i}(k):=\sqrt{Q(k)}\times random(n,1)$,rondom(n,1) represents the random function matrix column obeying Gaussian distribution.

Step 2: Calculate the predicted value ${\bar{x}}^{E}(k+1|k)$ of state x(k+1), and calculate the predicted value of measurement ${\bar{z}}^{E}(k+1|k)$ based on state predicted value z(k+1).

Step 3: The self-covariance matrix $P_{zz}^{E}(k+1|k)$ of measurement prediction error and the mutual covariance matrix $P_{xz}^{E}(k+1|k)$ of state prediction error and measurement prediction error are obtained.

Step 4: According to the covariance matrix of step 3, the gain matrix K(k+1) is obtained, and through the gain matrix update error, the state estimation members ${\hat{x}}_{i}^{E}(k+1|k+1)$ in the state estimation ensemble are obtained.

Step 5: According to step 4, the ensemble average ${\hat{x}}^{E}(k+1|k+1)$ and the estimation error covariance $P^{E}(k+1|k+1)$ of the estimated values of state x(k+1) are obtained.

**Note 3.1:** Review of algorithm advantages: the idea of the EnKF algorithm is simple and easy to implement. It uses the Monte Carlo method to generate an ensemble of states, and by obtaining more sampling points, as close as possible to the distribution of the real state, a better filtering effect can be obtained.

**Note 3.2:** Limitations of the EnKF algorithm: EnKF generates state ensemble by random sampling, which uses uncertain sampling, and the generated samples do not all conform to the distribution of real states. Therefore, it is difficult to approximate the real function and the real statistical characteristics of state variables, which will lead to the estimation error of state. Therefore, it is necessary to improve this random sampling method and find a sampling method that conforms to the real statistical characteristics of state variables as much as possible.

**Note 3.3:** The EnKF method approximates the best-predicted value and best-estimated value of the state by ensemble average. The disadvantage of this method is that the ensemble average is not necessarily the sample center of the ensemble, and it cannot reflect the true distribution of state variables. Since the ensemble average uses equal weights, the geometric center of the state is obtained, but the geometric center of the sample and the physical center of the sample are not necessarily coincident. Regarding the weight of each sample as the same, it will cause the lack of important information of the state variable, just as the effect of force on the centroid depends on the magnitude of the moment, the contribution of each sample point to the sample center is different. Therefore, it is necessary to improve this equal weight calculation method and find a weight calculation method that can reasonably describe the contribution of each sample.

Therefore, the selection of a sampling mechanism that can conform to the statistical characteristics of system state x(k), as well as the selection of appropriate weighting coefficients, become key issues to improve the effectiveness of EnKF in practical applications.

## 4. Unscented Kalman Filter Algorithm

Although EKF solves the situation that KF cannot solve the nonlinear problem [7], EKF lacks better approximation ability. Since EKF does not use the non-linear characteristics of the original non-linear function, it uses the method of non-linear approximation, discards the non-linear characteristics, and only uses the linear characteristics. The filter performance of its algorithm often degrades continuously with the increase in non-linearity, and it even diverges. For this reason, Julier et al. [8,9,10,11,12,13] introduced UKF to improve EKF. The idea is based on UT transformation, which is used to transfer the mean and covariance during one-step prediction. Therefore, according to Equation (7), on the basis that z(k+1) has been obtained. Based on the UKF method, the estimated value ${\hat{x}}^{U}(k+1|k+1)$ of state x(k+1) and the estimation error covariance matrix $P^{U}(k+1|k+1)\;$ will be derived. The specific algorithm steps are as follows:

Step 1: According to the estimator ${\hat{x}}^{U}(k|k)$ and covariance matrix $P^{U}(k|k)$ of the state at time k, a set of sample points $x_{i}(k|k)$ and corresponding weights $W_{i}$ are obtained.

Step 2: A set of new sample points $x_{i}^{U}\left(k+1|k\right)$ are obtained through nonlinear function $f(x_{i}(k|k))$, through the weight, the one-step prediction value ${\bar{x}}^{U}\left(k+1|k\right)$ of the state is obtained, and the error covariance matrix $P_{xx}^{U}\left(k+1|k\right)$ is calculated.

Step 3: Based on the predicted value $x_{i}^{U}\left(k+1|k\right)$ at time k+1, the measured predicted value $z_{i}^{U}\left(k+1|k\right)$ at time k+1 is obtained, ${\bar{z}}^{U}\left(k+1|k\right)$ is obtained by calculating and the self-covariance matrix $P_{zz}^{U}(k+1|k)$ and mutual covariance matrix $P_{xz}^{U}\left(k+1|k\right)$ are calculated.

Step 4: The gain matrix K(k+1) is calculated based on obtaining $P_{zz}^{U}(k+1|k)$ and $P_{xz}^{U}\left(k+1|k\right)$.

Step 5: Calculate the estimated value ${\hat{x}}^{U}(k+1|k+1)$ at time k+1 and the covariance matrix $P^{U}(k+1|k+1)$.

**Note 4.1:** UKF algorithm advantage comment: the algorithm operates normally, experimental analysis shows that the UKF can achieve a nonlinear second-order accuracy approximation close to Taylor expansion, and the filtering performance is superior to the first-order linear approximation of the EKF, thus improving the handling power for nonlinear problems.

**Note 4.2:** The defects in the selection of UKF parameters often cause the algorithm to fail to perform effectively: the main manifestation is that it is easy to appear negative definite phenomena in the three matrices $P_{xx}^{U}(k+1|k)$, $P_{zz}^{U}(k+1|k)$, and $P_{xx}^{U}(k+1|k+1)$. Analyses as below:

(1) The reasons of $P_{xx}^{U}(k+1|k),P_{zz}^{U}(k+1|k)$ might be negative definite:

In fact, for any $\theta \neq 0\in {\mathbb{R}}^{n\times 1}\;$, there is

(9) $$\begin{array}{ll} \theta^{T}P_{xx}^{U}\left(k+1|k\right)\theta & =\sum_{i=0}^{2n}W_{i}^{c}\theta^{T}\left[x_{i}\left(k+1|k\right)-{\bar{x}}^{U}\left(k+1|k\right)\right]\\ & \times {\left[x_{i}\left(k+1|k\right)-{\bar{x}}^{U}\left(k+1|k\right)\right]}^{T}\theta +\theta^{T}Q(k)\theta \end{array}$$

where, we let $r_{i}=\theta^{T}\left[x_{i}\left(k+1|k\right)-{\bar{x}}^{U}\left(k+1|k\right)\right]\in {\mathbb{R}}^{1}$, then

(10) $$\theta^{T}P_{xx}^{U}\left(k+1|k\right)\theta =\sum_{i=0}^{2n}W_{i}^{c}r_{i}^{2}+\theta^{T}Q\theta$$

Since matrix Q(k)≥0  is non-negative definite, thus, $\theta^{T}Q(k)\theta \geq 0\;$ in consideration of

(11) $$W_{i}^{c}=1/2(n+\lambda )>0;\;i=1,2,\ldots ,2n$$

So, there are

(12) $$\sum_{i=0}^{2n}W_{i}^{c}r_{i}^{2}=W_{0}^{c}r_{0}^{2}+\sum_{i=1}^{2n}W_{i}^{c}r_{i}^{2}$$

If $\sum_{i=0}^{2n}W_{i}^{c}r_{i}^{2}=W_{0}^{c}r_{0}^{2}+\sum_{i=1}^{2n}W_{i}^{c}r_{i}^{2}>0$, there will be $\sum_{i=1}^{2n}W_{i}^{c}r_{i}^{2}>-W_{0}^{c}r_{0}^{2}$.

So, whether $P_{xx}^{U}(k+1|k)$ is positive depends on the choice of $W_{0}^{c}$, if $W_{0}^{c}\geq 0$, then $P_{xx}^{U}(k+1|k)>0$, otherwise, there is no way to guarantee that matrix $P_{xx}^{U}(k+1|k)$ is positive definite.

In the same way, the analysis and results are similar for matrix $P_{zz}^{U}(k+1|k)$, which mainly depends on the value case of $W_{0}^{m}$.

(2) Analyze whether $P_{xx}^{U}(k+1|k+1)\;$ is positive definite. due to

(13) $$P_{xx}^{U}(k+1|k+1)=P_{xx}^{U}(k+1|k)-K(k+1)P_{zz}^{U}(k+1|k)K^{T}(k+1)$$

If $P_{zz}^{U}(k+1|k)$ is negative definite, because the gain matrix cannot be obtained

(14) $$K(k+1)=P_{xz}^{U}(k+1|k){\left[P_{zz}^{U}(k+1|k)\right]}^{-1}$$

This causes the algorithm to fail at this step, and thus causes the algorithm to fail.

(3) If $P_{zz}^{U}(k+1|k)\;$ is positive definite and $P_{xx}^{U}(k+1|k)\;$ is negative definite, the algorithm will fail in calculating the gain matrix.

This is because we want to make

(15) $$P_{xx}^{U}(k+1|k+1)>0$$

There must be

(16) $$P_{xx}^{U}(k+1|k)-K(k+1)P_{zz}^{U}(k+1|k)K^{T}(k+1)>0$$

which is

(17) $$P_{xx}^{U}(k+1|k)>K(k+1)P_{zz}^{U}(k+1|k)K^{T}(k+1)$$

Because of

(18) $$K(k+1)P_{zz}^{U}(k+1|k)K^{T}(k+1)>0$$

In fact, for any $\theta \neq 0\in {\mathbb{R}}^{n\times 1}\;$, there is

(19) $$\theta^{T}K(k+1)P_{zz}^{U}(k+1|k)K^{T}(k+1)\theta =\varphi P_{zz}^{U}(k+1|k)\varphi^{T}>0$$

(20) $$\;\varphi :=\theta^{T}K(k+1)$$

So, $K(k+1)P_{zz}^{U}(k+1|k)K^{T}(k+1)$ is positive definite, the $P_{xx}^{U}(k+1|k)$ must be positive definite.

From the above analysis, it can be seen that the problems of the UKF algorithm are caused by improper selection of weights in step 1. In actual simulation experiments, they are mainly caused by the negative values of $W_{0}^{m}$ and $W_{0}^{c}$. Therefore, selecting appropriate weights has become a bottleneck problem that affects the effective operation of UKF.

**Note 4.3:** Sigma sampling describes the statistical characteristics of the state: The UKF sampling is based on the estimation error covariance matrix $P^{U}(k|k)$ of state x(k), which is derived based on the statistical properties of estimation error ${\tilde{x}}^{U}(k|k)$ compared to the unconstrained random sampling of EnKF. However, when the dimension n is small, there are few sampling points, so sampling is difficult to be representative of the statistical characteristics of state x(k); when n is large, the obtained 2n + 1 sampling points are sparse relative to the n-dimensional space, and it is difficult to form coverage of the target characteristics.

Therefore, if the UKF sampling mechanism is combined with the EnKF random sampling mechanism, it will make up for the corresponding deficiencies of the two methods in the sampling mechanism. At the same time, if a better weight selection mechanism can be changed to overcome the shortcomings of UKF in sampling, it will not only overcome the bottleneck problem of frequent failure of UKF, but also greatly improve EnKF’s filtering performance that meets the statistical characteristics of actual problems.

## 5. Design of a Novel Kalman Filter

In order to overcome the respective shortcomings of UKF and EnKF, in this section, a novel data sampling driven Kalman filter is designed by combining the characteristic sampling of UKF and the random sampling of EnKF. This section first establishes a state sampling mechanism that combines UKF sampling and EnKF; second, establishes a weight selection method based on sampling ensembles; third, design a new Kalman filter design method combining UKF sampling and adaptive weight selection.

The previous analysis shows that the sampling randomness of EnKF is too strong, and the generation of random samples is not constrained by the statistical characteristics of random state variables. Although UKF is constrained by statistical characteristics, due to its own shortcomings, such as relying too much on parameter selection, the samples obtained are not representative in both low and high dimensions, so it is difficult to approach the statistical characteristics of its real state. Therefore, the new Kalman filter design method proposed in this section mainly solves the following two key problems:

(1) Establishing a large sample collection of objects that is suitable for the random characteristics of the objects and has a wider representation.

(2) Looking for a weight selection mechanism that avoids the failure of the algorithm.

### A Novel Data Sampling Driven Kalman Filter Is Designed by Combining the Characteristic Sampling of UKF and the Random Sampling of EnKF

The purpose of this section is to establish a new Kalman filter design method.

(1) Large sample generation and sampling mechanism of a new Kalman filter

1. Sigma sampling mechanism optimized based on UKF. In order to overcome the situation that the state variables in EnKF are not constrained by statistical characteristics. First, the state estimate ${\hat{x}}^{UE}(k|k)$ and the estimation error covariance matrix $P_{xx}^{UE}(k|k)$ at time k are known, and a set of sigma points is taken through the following sigma sampling mechanism of UKF

(21) $${\hat{x}}_{i}^{UE}(k|k)={\hat{x}}^{UE}(k|k),i=0$$

(22) $${\hat{x}}_{i}^{UE}(k|k)=x^{UE}(k|k)+\alpha {\sqrt{P_{xx}^{UE}(k|k)}}_{i},i=1,2,\cdots ,n$$

(23) $${\hat{x}}_{i}^{UE}(k|k)=x^{UE}(k|k)-\alpha {\sqrt{P_{{}^{xx}}^{UE}(k|k)}}_{i},i=n,n+1,\cdots ,2n$$

where, Equations (22) and (23) design parameters α, and α determines the dispersion of sample points; the parameters of the UKF are designed inside the square root sign, where the parameters are placed outside the square root to avoid the creation of negative values inside the square root. ${\sqrt{P_{xx}^{UE}(k|k)}}_{i}$ represents the i-th column vector after the square root of $P_{xx}^{UE}(k|k)$. The superscript “UE” in the equation represents the filtering result of the new Kalman filter.

2. EnKF random sampling large sample ensemble generation based on optimized UKF mechanism. Based on the optimized UKF sigma sampling mechanism established in 1, the sampling samples are expanded by using EnKF’s Monte Carlo random sampling mechanism at each obtained sigma sampling point.

First, for each sigma sampling point obtained from Equations (21)–(23)

(24) $${\hat{x}}_{i}^{UE}(k|k);i=0,\cdots ,n,n+1,\cdots ,2n$$

Using the EnKF Monte Carlo random sampling mechanism established by Equation (8), the sample expansion based on the sigma sampling point is obtained.

(25) $$\begin{array}{cc} X_{i}^{UE}(k|k)= & \{{\hat{\bar{x}}}_{ij}^{UE}(k|k)|{\hat{\bar{x}}}_{ij}^{UE}(k|k)\\ := & {\hat{x}}_{i}^{UE}(k|k)\pm r_{ij}(k);j=1,\cdots ,L_{i}\;;i=0,1,\cdots ,2n\} \end{array}$$

**Remark** **3.**
$L_{i}$ *is used to represent the number of columns that need to be increased for a single member in the collection. The value of* $L_{i}$ *is generally taken to be within 100.*

Where, $r_{ij}(k)\;$ is an arbitrary random perturbation, for example, a frequently chosen is $r_{ij}(k):=\sqrt{Q(k)}\times random(n,1)$.

Integrating Equation (25), the sample ensemble used to describe the state statistical properties of state ${\hat{x}}^{UE}(k|k)$ was obtained

(26) $$X^{UE}(k|k)=\overset{2n}{\underset{i=0}{\cup }}X_{i}^{UE}(k|k)$$

**Remark** **4.**
$X^{UE}(k|k)$ *represents the ensemble of States and* $X_{i}^{UE}(k|k)$ *represents the ensemble of individual members.*

Where, the number of samples in sample ensemble $X^{UE}(k|k)$ is $|X^{UE}(k|k)|=\sum_{i=0}^{2n}L_{i}$.

(2) State x(k+1) One-step prediction estimation and estimation error covariance matrix based on a large sample ensemble.

1. Based on the Equation (25), the state prediction at k+1 for each member of the ensemble of time k is computed

(27) $${\hat{\bar{x}}}_{ij}^{UE}(k+1|k)=f({\hat{\bar{x}}}_{ij}^{UE}(k|k)),i=0,1,\cdots ,2n;j=1,2,\cdots ,L_{i}$$

2. Calculate the confidence weight of each predicted value ${\hat{\bar{x}}}_{ij}^{UE}(k+1|k)$.

(28) $$\left\{ \begin{array}{l} \left\{{\hat{\bar{x}}}_{ij}^{UE}(k+1|k);i=0,\cdots ,2n;j=1,2,\cdots ,L_{i}\right\}\\ \forall l,d({\hat{\bar{x}}}_{l}^{UE}(k+1|k))\\ =\sum_{i=0}^{2n}\sum_{j=1}^{L_{i}}{\left\|{\hat{\bar{x}}}_{l}^{UE}(k+1|k)-{\hat{\bar{x}}}_{ij}^{UE}(k+1|k)\right\|}_{2};l=01,\cdots ,ij,\cdots ,2nL_{i}\\ l_{0}=\arg\min_{l}\left\{d({\hat{\bar{x}}}_{l}^{UE}(k+1))\right\} \end{array}\right.$$

(29) $$d_{ij}^{UE}(k+1|k)={\left\|{\hat{\bar{x}}}_{l_{0}}^{UE}(k+1|k)-{\hat{\bar{x}}}_{ij}^{UE}(k+1|k)\right\|}_{2}$$

(30) $$D_{ij}^{UE}(k+1|k)=\frac{1/d_{ij}^{UE}(k+1|k)}{\sum_{i=0}^{2n}\sum_{j=1}^{L_{i}}1/d_{ij}^{UE}(k+1|k)},ij\neq l_{0}$$

where, when ${\hat{\bar{x}}}_{ij}^{UE}(k+1|k)={\hat{\bar{x}}}_{l_{0}}^{UE}(k+1|k)$, $D_{l_{0}}^{UE}(k+1|k)=0$. Here the weight at the centroid, we temporarily set it to 0. The reason is to avoid calculating the distances of the centroids themselves and, in the following processing, to reassign weights to the centroids.

3. Calculate the one-step forecast estimate of x(k+1).

(31) $${\hat{x}}^{UE}(k+1|k)=(1-\beta )\sum_{i=0}^{2n}\sum_{j=1}^{L_{i}}D_{ij}^{UE}(k+1|k){\hat{\bar{x}}}_{ij}^{UE}(k+1|k)+\beta {\hat{\bar{x}}}_{l_{0}}^{UE}(k+1|k)$$

Calculate the state prediction estimation error of x(k+1)

(32) $${\tilde{x}}^{UE}(k+1|k)=x(k+1)-{\hat{x}}^{UE}(k+1|k)$$

**Note 5.1.1:** when calculating the weight at Equation (29), the weight at the centroid is temporarily set to 0. Here, the weight of the centroid and other members is redistributed. 0<β<1, β represents the contribution of the centroid to the whole ensemble, and the contribution rate of the centroid in the whole filtering process can be adjusted through β. According to the principle of exponential filtering, in general we take β=0.5, which, in experiments, can be increased exponentially, modulating the contribution of the centroid.

4. Obtain the one-step prediction error covariance matrix of x(k+1)**.**

(33) $$\begin{array}{l} P_{xx}^{UE}(k+1|k)\\ =\sum_{i=0}^{2n}\sum_{j=1}^{L_{i}}D_{ij}^{UE}({\hat{\bar{x}}}_{ij}^{E}(k+1|k)-{\hat{x}}^{UE}(k+1|k)){\left({\hat{\bar{x}}}_{ij}^{E}(k+1|k)-{\hat{x}}^{UE}(k+1|k)\right)}^{T} \end{array}$$

(3) One step prediction estimation and prediction estimation error covariance matrix of measurement z(k+1).

1. Based on Equation (27), calculate the measured predicted value of each member in the ensemble at time k at time k+1.

(34) $${\hat{\bar{z}}}_{ij}^{UE}(k+1|k)=h({\hat{\bar{x}}}_{ij}^{UE}(k+1|k));i=0,1,\cdots ,2n;j=1,2,\cdots ,L_{i}$$

2. Calculate the confidence weight of each measured predicted value ${\hat{\bar{z}}}_{ij}^{UE}(k+1|k)$.

(35) $$\left\{ \begin{array}{l} \left\{{\hat{\bar{z}}}_{ij}^{UE}(k+1|k);i=0,\cdots ,2n;j=1,2,\cdots ,L_{i}\right\}\\ \forall l,d({\hat{\bar{z}}}_{l}^{UE}(k+1|k))\\ =\sum_{i=0}^{2n}\sum_{j=1}^{L_{i}}{\left\|{\hat{\bar{z}}}_{l}^{UE}(k+1|k)-{\hat{\bar{z}}}_{ij}^{UE}(k+1|k)\right\|}_{2};l=01,\cdots ,ij,\cdots ,2nL_{i}\\ l_{1}=\arg\min_{l}\left\{d({\hat{\bar{z}}}_{l}^{UE}(k+1|k))\right\} \end{array}\right.$$

(36) $$d_{zij}^{UE}(k+1|k)={\left\|{\hat{\bar{z}}}_{l_{1}}^{UE}(k+1|k)-{\hat{\bar{z}}}_{ij}^{UE}(k+1|k)\right\|}_{2}$$

(37) $$D_{zij}^{UE}(k+1|k)=\frac{1/d_{zij}^{UE}(k+1|k)}{\sum_{i=0}^{2n}\sum_{j=1}^{L_{i}}1/d_{zij}^{UE}(k+1|k)},ij\neq l_{1}$$

where, when ${\hat{\bar{z}}}_{ij}^{UE}(k+1)={\hat{\bar{z}}}_{l_{1}}^{UE}(k+1|k)$, $D_{l_{1}}^{UE}(k+1|k)=0$. Similarly, when measuring the weight at the centroid of the prediction ensemble, we temporarily set it to 0. The reason is to avoid calculating the distances of the centroids themselves and, in the following processing, to reassign weights to the centroids.

3. Calculate the one-step prediction estimate of z(k+1).

(38) $${\hat{z}}^{UE}(k+1|k)=(1-\beta )\sum_{i=0}^{2n}\sum_{j=1}^{L_{i}}D_{zij}^{UE}(k+1|k){\hat{\bar{z}}}_{ij}^{UE}(k+1|k)+\beta {\hat{\bar{z}}}_{l_{1}}^{UE}(k+1|k)$$

Calculate the measurement prediction error between the measured predicted value and the real value at *k* + 1 time.

(39) $${\tilde{z}}^{UE}(k+1|k)=z(k+1)-{\hat{z}}^{UE}(k+1|k)$$

4. Obtain the one-step prediction error covariance matrix of z(k+1).

(40) $$\begin{array}{l} P_{zz}^{UE}(k+1|k)\\ =\sum_{i=0}^{2n}\sum_{j=1}^{L_{i}}D_{zij}^{UE}({\hat{\bar{z}}}_{ij}^{UE}(k+1|k)-{\hat{z}}^{UE}(k+1|k)){\left({\hat{\bar{z}}}_{ij}^{UE}(k+1|k)-{\hat{z}}^{UE}(k+1|k)\right)}^{T} \end{array}$$

(4) Mutual covariance matrix between state prediction error and measurement prediction error.

(41) $$\begin{array}{cc} P_{xz}^{UE}(k+1|k)= & \sum_{i=0}^{2n}\sum_{j=1}^{L_{i}}D_{ij}^{UE}({\hat{\bar{x}}}_{ij}^{E}(k+1|k)-{\hat{x}}^{UE}(k+1|k))\\ & \times D_{zij}^{UE}{\left({\hat{\bar{z}}}_{ij}^{UE}(k+1|k)-{\hat{z}}^{UE}(k+1|k)\right)}^{T} \end{array}$$

(5) Design of a novel Kalman filter.

1. Calculate the estimated value ${\hat{x}}_{i}^{UE}(k+1|k+1)$ of each member in the ensemble at k+1.

(42) $${\hat{\bar{x}}}_{ij}^{UE}(k+1|k+1)={\hat{\bar{x}}}_{ij}^{UE}(k+1|k)+K(k+1)[z(k+1)-h({\hat{\bar{x}}}_{ij}^{UE}(k+1|k))]$$

2. Calculate the confidence weight of each estimate ${\hat{x}}_{i}^{UE}(k+1|k+1)$.

(43) $$\left\{ \begin{array}{l} \left\{{\hat{\bar{x}}}_{ij}^{UE}(k+1|k+1)\right\};i=0,\cdots ,2n;j=1,2,\cdots ,L_{i}\\ \forall l,d({\hat{\bar{x}}}_{l}^{UE}(k+1|k+1))\\ =\sum_{i=0}^{2n}\sum_{j=1}^{L_{i}}{\left\|{\hat{\bar{x}}}_{l}^{UE}(k+1|k+1)-{\hat{x}}_{ij}^{UE}(k+1|k+1)\right\|}_{2};l=01,\cdots ,ij,\cdots ,2nL_{i}\\ l_{2}=\arg\min_{l}\left\{d({\hat{\bar{x}}}_{l}^{UE}(k+1|k+1))\right\} \end{array}\right.$$

(44) $$d_{ij}^{UE}(k+1|k+1)={\left\|{\hat{x}}_{l_{2}}^{UE}(k+1|k+1)-{\hat{x}}_{i}^{UE}(k+1|k+1)\right\|}_{2}$$

(45) $$D_{ij}^{UE}(k+1|k+1)=\frac{1/d_{ij}^{UE}(k+1|k+1)}{\sum_{i=0}^{2n}\sum_{j=1}^{L_{i}}1/d_{ij}^{UE}(k+1|k+1)},ij\neq l_{2}$$

where, when ${\hat{\bar{x}}}_{ij}^{UE}(k+1|k+1)={\hat{\bar{x}}}_{l_{2}}^{UE}(k+1|k+1)$, $D_{l_{2}}^{UE}(k+1|k+1)=0$. Similarly, the weight at the centroid is temporarily set to 0, the reason is to avoid calculating the distances of the centroids themselves and, in the following processing, to reassign weights to the centroids.

3. Calculate the estimate of x(k+1).

(46) $$\begin{array}{l} {\hat{x}}^{UE}(k+1|k+1)\\ =(1-\beta )\sum_{i=0}^{2n}\sum_{j=1}^{L_{i}}D_{ij}^{UE}(k+1|k+1){\hat{\bar{x}}}_{ij}^{UE}(k+1|k+1)+\beta {\hat{\bar{x}}}_{l_{2}}^{UE}(k+1|k+1) \end{array}$$

Calculate the error of the state estimate

(47) $${\tilde{x}}^{UE}(k+1|k+1)=x(k+1)-{\hat{x}}^{UE}(k+1|k+1)$$

(6) Calculate the gain matrix.

According to the orthogonal principle

(48) $$E\left\{{\tilde{x}}^{UE}(k+1|k+1){\left[{\tilde{z}}^{UE}(k+1|k)\right]}^{T}\right\}=0$$

It can be obtained from Equations (32),(39) and (47).

(49) $$\begin{array}{l} E\left\{\left[x(k+1)-{\hat{x}}^{UE}(k+1|k+1)\right]{\left[z(k+1)-{\hat{z}}^{UE}(k+1|k)\right]}^{T}\right\}\\ =E\left\{{\tilde{x}}_{k+1}{\tilde{z}}_{{}_{k+1}}^{T}-K{\tilde{z}}_{k+1}{\tilde{z}}_{{}_{k+1}}^{T}+Kv_{k+1}v_{{}_{k+1}}^{T}\right\}\\ =E\left\{P_{xz}(k+1|k)-KP_{zz}(k+1|k)+KR\right\}\\ =0 \end{array}$$

Then there

(50) $$P_{{}_{xz}}^{UE}(k+1|k)-KP_{{}_{zz}}^{UE}(k+1|k)+KR=0$$

(51) $$P_{{}_{xz}}^{UE}(k+1|k)-K\left[P_{{}_{zz}}^{UE}(k+1|k)+R\right]=0$$

Then the gain matrix is

(52) $$K(k+1)=P_{{}_{xz}}^{UE}(k+1|k){\left[P_{{}_{zz}}^{UE}(k+1|k)+R\right]}^{-1}ax(k+1)$$

(7) Calculate the estimation error covariance matrix of state x(k+1)

(53) $$\begin{array}{cc} P^{UE}(k+1|k+1) & =\sum_{i=0}^{2n}\sum_{i=1}^{L_{i}}D_{ij}^{UE}({\hat{\bar{x}}}_{ij}^{UE}(k+1|k+1)-{\hat{x}}^{UE}(k+1|k+1))\\ & \times ({\hat{\bar{x}}}_{ij}^{UE}(k+1|k+1)-{\hat{x}}^{UE}{\left(k+1|k+1\right)}^{T} \end{array}$$

## 6. System Performance Analysis

This section mainly analyzes the methods in Section 5.

### 6.1. The Shortcomings of UKF and EnKF Sampling Mechanisms

It is known from the analyses in Section 3 and Section 4 that both EnKF and UKF have their own insufficiencies in their sampling mechanisms. The way EnKF produces state ensembles is a stochastic sampling, hard to approximate the true function and hard to approximate the true statistical properties of state variables. Although UKF sampling method can represent the statistical characteristics of the state, the number of samples is relatively small.

In response to the above insufficiencies, a new sampling mechanism was established for the novel Kalman filter design method of Section 5, mainly to solve the problem that EnKF cannot be constrained by the true statistical properties of the states when it is randomly sampled by Monte Carlo, and to obtain more representative sample points.

### 6.2. Weight Selection Analysis of UKF and EnKF

It can be seen from the analysis in Section 3 and Section 4 that there are defects in the weight selection of EnKF and UKF. The weight distribution of EnKF regards the weight of each sample as the same, which will cause the loss of important information of state variables. Just as the effect of force on the center of moment depends on the size of moment, the contribution of each sample point to the center of sample is different. The weight selection of UKF will cause the non-positive definite of error covariance matrix, resulting in the failure of algorithm operation. In view of the above problems, the method in Section 5 avoids the lack of information caused by EnKF weight. It overcomes the problem of algorithm failure in UKF operation.

### 6.3. Improvement of Filter Performance

For EnKF, we use the UKF sampling mechanism affected by the statistical characteristics and the statistical characteristics of state x(k), so that EnKF can approach the real statistical characteristics of system state variables when sampling. In terms of weight selection, the distribution of weight coefficients is more reasonable, which makes the state closer to the real distribution and avoids the loss of information.

For UKF, based on the statistical characteristics of UKF, the dense sampling of EnKF sampling mechanism is mainly reflected in that the state prediction is more accurate, the approximate error is reduced, and the approximate error of measurement is reduced. The combination of two points improves the estimation accuracy. Moreover, the selection of weight avoids the failure of the algorithm and enhances the stability of the algorithm.

### 6.4. Error Analysis

In order to analyze the feasibility of the method proposed in this article, we will conduct error analysis from the following aspects:

1. Analysis of the approximation error of the state

Define ${\hat{\bar{x}}}_{i}(k+1|k)$ as the i-th state component of the predicted value of x(k) at time k+1, and $\Delta e_{i}^{E}(k)$ is the approximation error between the predicted state value of EnKF at time *k* and the true value.

(54) $$\Delta e_{i}^{E}(k)=|\left({\hat{\bar{x}}}_{i}^{E}(k+1|k)-x_{i}(k+1)\right)|,i=1,2,\cdots ,n$$

Then the approximation error of state prediction is

(55) $$Error1=\frac{1}{Nn}\sum_{k=1}^{N}\sum_{i=1}^{n}\Delta e_{i}^{E}(k)$$

${\hat{\bar{x}}}_{i}^{UE}(k+1|k)\;$ is defined as the i-th state component of the predicted value at k+1 time of x(k), and $\Delta e_{i}^{UE}(k)\;$ is the approximation error between the predicted value and the real value of new Kalman filter at k+1 time.

(56) $$Error2=\frac{1}{Nn}\sum_{k=1}^{N}\sum_{i=1}^{n}\Delta e_{i}^{UE}(k)$$

We analyze the predicted value of the state and compare the results of Equations (55) and (56). From the theoretical analysis, it should be that the method proposed in this paper reduces the approximation error between the predicted value and the real value, so the estimation error also decreases, and has a better filtering effect.

2. Analysis of estimation error of state:

$x_{i}(k)$ is defined as the *i*-th state component of x(k), and $\Delta F_{i}(k)$ is the error between the estimated state value and the real value.

(57) $$\Delta F_{i}(k)={\hat{x}}_{i}(k|k)-x_{i}(k)$$

(58) $$MSE_{\Delta F}=\frac{1}{nN}\sum_{k=1}^{N}\sum_{i=1}^{n}{\left(\Delta F_{i}(k)\right)}^{2}$$

We calculate the mean square error between the estimated value and the real value of the state through Equations (57) and (58), and compare and analyze them through experiments. If the approximation error decreases and the estimation error decreases accordingly, it is proved that the error result of the estimation value is consistent with the approximation error. The approximation error is small and the estimation error is small, which can show the effectiveness of the method in this paper.

## 7. Simulation Experiment

### 7.1. Simulation Experiment One: Single Dimensional Simulation

Consider the following one-dimensional nonlinear system:

(59) $$x(k+1)=0.5x(k)+2.5x(k)/(1+x^{2}(k))+8\cos(1.2k)+w(k)$$

(60) $$z(k+1)=\gamma x^{2}(k+1)+v(k+1)$$

where, w(k), v(k+1), are all Gaussian white noise sequences obeying a distribution of N(0,0.01), with initial value m, assuming initial estimate $\hat{x}(0|0)=1.1$, initial estimate error covariance P(0∣0)=0.1, and γ as parameters that regulate the degree of nonlinearity. Here are the comparative trials under different methods:

**Remark** **5.** * The row of the table represents the sampling method used, and the column of the table represents the weight method used. The improvement in the row represents the comparison results of different weights selected by the same sampling method, and the improvement in the column represents the comparison results of different samples selected by the same weight method. The improved cross Italic part represents the results of the comparison between the weight and sampling method in this paper and the original EnKF.*

**Figure 1:**
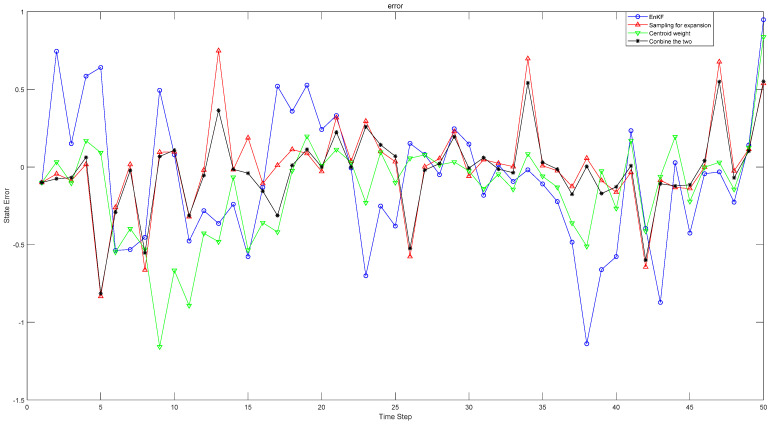
The error between the estimated value and the true value of the state.

Figure 1 shows the comparison between the errors of estimated value and real value in one-dimensional simulation experiment when γ=0.05. The methods in the figure are the original EnKF, the EnKF changing the sampling method, the EnKF changing the weight selection and the new Kalman filter method combining the two methods finally adopted in this paper. Table 1 shows the comparison results under different parameters, different weights and different sampling methods. When γ=0.05, under the original weight, the approximation error of the original sampling EnKF method is 0.3445, and the approximation error of the expansion sampling method is 0.2923. In contrast, the EnKF of the expansion sampling method is 15.15% higher than the original. Under the original sampling method, the approximation error of the confidence weight is 0.2824, which is 18.02% higher than the original weight. The approximation error of the method proposed in this paper is 0.2807, which is 18.52% higher than the original EnKF. Compared with the original weight, the EnKF method of capacity expansion sampling is improved by 3.97%, and compared with the EnKF method of confidence weight, the EnKF method of original sampling is improved by 0.6%. In the estimation error analysis in Table 2, the original weight is increased by 21.97% compared with the original EnKF by using the expansion sampling method, the original sampling is increased by 44.9% compared with the original EnKF by using the confidence weight, and the centroid weight is increased by 46.71% compared with the original EnKF by using the centroid weight expansion sampling method. In addition, the effect of changing both sampling and weight is better than that of changing only one method. With the reduction of approximation error in Table 1, the corresponding estimation error in Table 2 is also reduced, and the filtering effect is greatly improved. In Table 1 and Table 2, with the change of parameters, the nonlinearity of the system is also changing. When the nonlinearity is enhanced, the method proposed in this paper still has good results. Through experiments, we verify the feasibility of our method in one-dimensional system.

### 7.2. Simulation Experiment Two: Multidimensional Simulation

**Figure 2:**
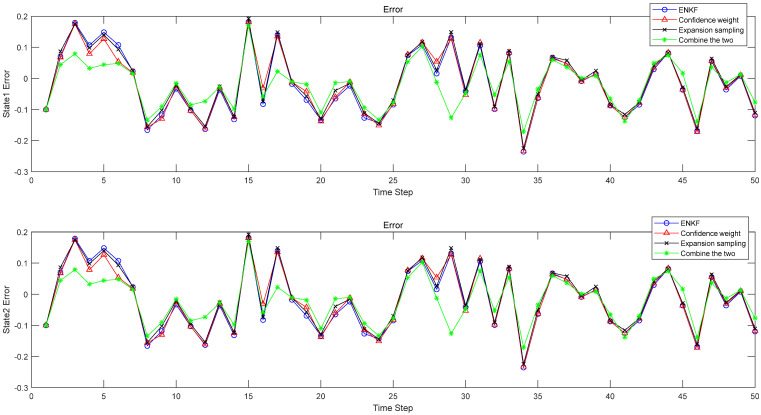
The error between the estimated value and the true value of the state.

Consider the following two-dimensional strongly nonlinear system:

(61) $$x(k+1)=\left\{ \begin{array}{l} \cos(\frac{1}{20}x_{1}(k))+\frac{1}{20}x_{2}(k)+w_{1}(k)\\ 2x_{1}^{2}(k)e^{-x_{1}(k)x_{2}(k)}+w_{2}(k) \end{array}\right.$$

(62) $$z(k+1)=\left\{ \begin{array}{l} \sin(\alpha x_{1}(k+1))+x_{2}(k+1)+v_{1}(k+1)\\ \cos(\beta x_{2}(k+1)+\gamma x_{1}(k+1)x_{2}(k+1))+v_{2}(k+1) \end{array}\right.$$

where, w(k), v(k+1), are all Gaussian white noise sequences obeying a distribution of N(0,0.01), assuming an initial true value of $x(0|0)={\left[1,1\right]}^{T}$, an initial estimate of $\hat{x}(0|0)={\left[1.1,1\right]}^{T}$ with initial estimation error covariates P(0∣0)=diag([0.1,0.1]), and α,β,γ is a parameter that regulates the degree of nonlinearity in the nonlinear system. The original EnKF method was contrasted with different methods separately, and the experimental results are shown below:

Figure 2 shows the comparison results of the errors of four methods when α=0.5,β=0.5,γ=1. Table 3, Table 4, Table 5 and Table 6 show the results when β=0.5 and γ=1 are constants and the nonlinearity of the system is changed by α. When α=0.5,β=0.5,γ=1, in Table 3, the approximation error of state $x_{1}$ in the original EnKF method is 0.1064, and the approximation error of the expansion sampling method is 0.1056. In contrast, the EnKF of the expansion sampling method is increased by 0.75% compared with the original. Under the original sampling method, the approximation error of the confidence weight is 0.1055, which is 0.85% higher than the original weight. The approximation error of the method proposed in this paper is 0.1050, which is 1.32% higher than the original EnKF. In the estimation error analysis in Table 4, under the original weight the expansion sampling method is 5.85% higher than the original EnKF; under the original sampling, the confidence weight is 6% higher than the original EnKF, and the centroid weight expansion sampling method is 45.44% higher than the original EnKF. As can be seen from the table, the effect of improving the two methods is better than changing only one. The experimental results show that the approximation error is reduced, the corresponding estimation error is also reduced, and the filtering effect is significantly improved. Similarly, in Table 5 and Table 6, the status $x_{2}$ has the same effect. Moreover, as can be seen from Table 3, Table 4, Table 5 and Table 6, with the enhancement of nonlinearity, the filtering effect of the method proposed in this paper is still very good. This experiment proves that the estimation error can be reduced when the approximation error is reduced. By reducing the approximation error and estimation error, the filtering accuracy is improved, and the feasibility of this method is verified.

## 8. Summary and Prospect

This paper improves the performance of Kalman filter for nonlinear system, contains the respective advantages of UKF and EnKF, overcomes the shortcomings of UKF and EnKF and establishes a new design method of Kalman filter based on sampling data drive. Firstly, a new sampling mechanism is proposed. Based on the sigma sampling with UKF statistical constraints, the sampling mechanism performs random sampling similar to EnKF around each sampling point, so as to obtain the large sample data ensemble of the characteristics of the system variables to be estimated. It overcomes the shortcomings of UKF and EnKF in the sampling mechanism. Secondly, a sample weight selection and assignment mechanism based on the centroid of data set is established, which overcomes the defects of UKF and EnKF in weight selection. Thirdly, combining the advantages of the two methods, a new Kalman filter driven by large data sample ensemble is established. Finally, through computer numerical simulation experiments, via different parameters, the best estimates under different conditions are obtained, which verify the effectiveness of the method in this paper.

Prospect: Although the method proposed in this paper solves the defects of UKF and EnKF, it is still insufficient. These sampling mechanisms are based on the assumption that the state to be estimated is normal distribution, but the state to be estimated is often a non-Gaussian variable that is difficult to conform to the normal distribution. How to establish a sampling mechanism that conforms to the real characteristics is the direction that still requires hard work. For non-Gaussian systems, further research is needed.

## Figures and Tables

**Table 1:** State approximation error contrasts.

	Sampling	Parameter	Original Sampling	Expansion Sampling	Improve
Weight					
Equal weighting		*γ* = 5	0.4588	0.4359	4.99%
Confidence weights		*γ* = 5	0.3861	0.3823	0.98%
Improvement		-	15.85%	16.43%	*16.67%*
Equal weighting		*γ* = 5	0.3292	0.2596	21.14%
Confidence weights		*γ* = 5	0.2570	0.2561	0.35%
Improvement		-	21.93%	1.35%	*22.2%*
Equal weighting		*γ* = 5	0.3445	0.2923	15.15%
Confidence weights		*γ* = 5	0.2824	0.2807	0.6%
Improvement		-	18.02%	3.97%	*18.52%*

**Table 2:** Mean square error contrasts.

	Sampling	Parameter	Original Sampling	Expansion Sampling	Improve
Weight					
Equal weighting		*γ* = 5	0.2816	0.2608	7.4%
Confidence weights		*γ* = 5	0.2508	0.2142	14.59%
Improvement		-	10.94%	17.87%	*23.93%*
Equal weighting		*γ* = 5	0.1881	0.0866	53.97%
Confidence weights		*γ* = 5	0.1217	0.0664	45.44%
Improvement		-	35.30%	23.32%	*64.70%*
Equal weighting		*γ* = 5	0.2376	0.1854	21.97%
Confidence weights		*γ* = 5	0.1309	0.1266	3.28%
Improvement		-	44.9%	31.71%	*46.71%*

**Table 3:** Approximating error contrasts of *x*_1_.

		Parameter	Original Sampling	Expansion Sampling	Improve
	**Sampling**	α	*x*_1_	*x*_1_	*x*_1_
**Weight**					
Equal weighting		α = 0.5	0.1064	0.1056	0.75%
Confidence weights		α = 0.5	0.1055	0.1050	0.47%
Improvement		-	0.85%	0.57%	*1.32%*
Equal weighting		α = 0.5	0.0893	0.0861	3.58%
Confidence weights		α = 0.5	0.0858	0.0810	5.59%
Improvement		-	3.91%	5.92%	*9.2%*
Equal weighting		α = 0.5	0.1054	0.1039	1.42%
Confidence weights		α = 0.5	0.1039	0.1029	0.96%
Improvement		-	1.42%	0.96%	*2.3%*

**Table 4:** Mean square error contrasts of *x*_1_.

		Parameter	Original Sampling	Expansion Sampling	Improve
	**Sampling**	α	*x*_1_	*x*_1_	*x*_1_
**Weight**					
Equal weighting		α = 0.5	0.0108	0.0101	5.85%
Confidence weights		α = 0.5	0.0101	0.0059	41.96%
Improvement		-	6%	29.67%	*45.44%*
Equal weighting		α = 0.5	0.0074	0.0070	5.57%
Confidence weights		α = 0.5	0.0069	0.0028	59.06%
Improvement		-	7.57%	59.93%	*62.16%*
Equal weighting		α = 0.5	0.0129	0.0125	3.08%
Confidence weights		α = 0.5	0.0115	0.0050	56.52%
Improvement		-	10.85%	60.3%	*61.52%*

**Table 5:** Approximating error contrasts of *x*_2_.

		Parameter	Original Sampling	Expansion Sampling	Improve
	**Sampling**	α	*x*_2_	*x*_2_	*x*_2_
**Weight**					
Equal weighting		α = 0.5	0.1850	0.1832	0.97%
Confidence weights		α = 0.5	0.1616	0.1612	0.25%
Improvement		-	12.65%	12%	*12.86%*
Equal weighting		α = 0.5	0.1649	0.1629	1.21%
Confidence weights		α = 0.5	0.1634	0.1346	17.63%
Improvement		-	0.91%	17.37%	*18.37%*
Equal weighting		α = 0.5	0.1983	0.1953	1.5%
Confidence weights		α = 0.5	0.1963	0.1535	21.8%
Improvement		-	1%	21.4%	*22.59%*

**Table 6:** Mean square error contrasts of *x*_2_.

		Parameter	Original Sampling	Expansion Sampling	Improve
	**Sampling**	α	*x*_2_	*x*_2_	*x*_2_
**Weight**					
Equal weighting		α = 0.5	0.0095	0.0088	7.13%
Confidence weights		α = 0.5	0.0086	0.0062	35.41%
Improvement		-	9.68%	7.13%	*34.69%*
Equal weighting		α = 0.5	0.0122	0.0123	−0.15%
Confidence weights		α = 0.5	0.0074	0.0073	1.94%
Improvement		-	39.25%	40.51%	*40.43%*
Equal weighting		α = 0.5	0.0131	0.0093	29.09%
Confidence weights		α = 0.5	0.0076	0.0067	11.18%
Improvement		-	42.21%	27.61%	*48.67%*
